# 

**Published:** 1888-12

**Authors:** 


THE BUFFALO MEDICAL AND SURGICAL ASSOCIATION will hold its regular monthly meeting at the Mechanics' Institute, No. 9 West Mohawk street, Tuesday evening, December 4th, at 8.30 o'clock.

THE SOUTHERN SURGICAL AND GYNECOLOGICAL ASSOCIATION will hold its first annual meeting in Birmingham, Ala., commencing Tuesday, December 4, 1888, and lasting three days. It will be remembered that this meeting was postponed from September, owing to the quarantine against yellow fever. Dr. W. E. B. Davis, of Birmingham, is the secretary, and the programme includes a number of papers upon interesting subjects.

The next Annual Meeting of the American Medical Association will be held in Newport, R. I., commencing Tuesday, June 25, 1889, and continuing four days. The change of date from the first to the last Tuesday of June, was made imperative by reason of the fact that no adequate hotel accommodations could be provided for the delegates and members on the date first named. The Rhode Island State Medical Society has already taken steps to coöperate with the committee of arrangements with a view to make the meeting both successful and pleasant. Newport celebrates *the 250th anniversary* of its settlement on the same date.

### BOOKS AND PAMPHLETS RECEIVED.

Treatise on the Diseases of Women. For the use of students and practitioners. By Alexander J. Skene, M. D., Professor of Gynecology in the Long Island College Hospital, Brooklyn, N. Y., etc., etc. With 261 engravings and nine chromo-lithographs. 8vo. pp. xiv., 956. New York: D. Appleton & Co. 1888.

The Life Insurance Examiner. A Practical Treatise upon Medical Examinations for Life Insurance. By Charles E. Stillman, M. S., M. D., Medical Examiner for the Mutual Life Insurance Company for the General Agency of the City of New York, etc., etc. Large 8vo, pp. 188; 16, viii. New York: The Spectator Company, 16 Dey street. 1888.

A Manual of Dietetics for Physicians, Mothers and Nurses. By W. B. Pritchard, M. D., New York City, Price, fifty cents. Published by the Dietetic Publishing Company, 115 Fulton street, New York.

Medical Diagnosis. A Manual of Clinical Methods. By J. Graham Brown, M. D., Fellow of the Royal College of Physicians of Edinburgh, late Senior President of the Royal Medical Society of Edinburgh. Second edition. Illustrated. New York: E. B. Treat, 171 Broadway. 1888.

Enterostomy for Acute Intestinal Obstruction. By B. Farquhar Curtis, M. D., Attending Surgeon to St. Luke's Hospital, New York. Author's reprint from the *Medical Record*, September 1, 1888.

Why Electrolytic Treatment of Stricture does not Succeed in all Hands. By G. C. H. Meier, M. D., Member of the New York State Medical Association. E. P. Cody & Co., New York. Author's reprint from the *International Journal of Surgery and Antiseptics*, October, 1888.

The Treatment of Peritonitis by Abdominal Section. Some illustrative cases. By L. S. McMurtry, A. M., M. D., formerly Professor of Anatomy in the Kentucky School of Medicine, etc. Author's reprint from the *Journal of the American Medical Association.*

A Case of Typhlitis, with Double Perforation of the Cecum and Peritonitis, in which Laparatomy and Suture of the Gut were followed by Recovery. By L. S. McMurtry, A. M., M. D. Read in the Section on Surgery at thirty-ninth annual meeting of the American Medical Association, May, 1888. Author's reprint from the *Journal of the American Medical Association*, July 7, 1888.

Consequences of Acute Suppuration of the Middle Ear, with Special Reference to Opening the Mastoid. By A. R. Baker, M. D. Author's reprint from Transactions Ohio State Medical Society, 1888.

Cataract Extractions, with only the Eye operated upon Closed by Adhesive Strips; the other Eye left Open for the Guidance of the Patient. By Julian J. Chisholm, M. D., Professor of Eye and Ear Surgery in the University of Maryland, etc. Author's reprint from the *Journal of the American Medical Association*, November 3, 1888.

The Great Value of a 0.25 D. Cylinder in the Relief of Headache and Eye Pains. By Julian J. Chisholm, M. D. Author's reprint from *Jour. Am. Med. Association*, Oct. 27, 1888.

Eczema: Its Treatment. Dr. Albert E. Carrier, M. D., Professor of Dermatology in the Detroit College of Medicine. Author's reprint from *The Physician and Surgeon*, November.

Subglottic Laryngeal Tumor. By E. Fletcher Ingals, M. D. Author's reprint from *The Medical News*, November 3, 1888.

Report on Hydrophobia. By Chas. W. Dulles, M. D. Author's reprint from the Transactions of the Medical Society of the State of Pennsylvania, June, 1888.

Phenacetine-Bayer (Para-Acetphenetidine). The New Antipyretic and Anti-neuralgic. Manufactured by the Farbenfabriken, formerly Friedr. Bayer Co., Elberfeld. W. H. Schieffelin & Co., Sole Licensees and Sole Agents for the United States.

Inebriate Asylums and their Work. T. D. Crothers, M. D., Superintendent Walnut Lodge, Hartford, Conn., etc. Part of a lecture before the Y. M. C. A., at Toronto, October 2, 1888. Author's reprint.

Zur Behandlung der Gebärmutterzerreissung während der Geburt von Prof. Dr. Leopold. Dresden. (Seperatabdruck a. d. Verhandl. deutschen Gesellsch. für Gynecologie, 1888.)

The Contagiousness of Phthisis (Tubercular Pulmonitis). By Lawrence Flick, M. D., Philadelphia. Reprinted from the Transactions of the Medical Society of the State of Pennsylvania, June, 1888.

The Constitution and By-Laws, with the Officers and Members for 1888–9, of the American Pediatric Society, organized in Washington, D. C., September 18, 1888. Reprinted from the Archives of Pediatrics.

Mineral and Thermal Springs of California. By W. F. McNutt, M. D., M. R. C. S., etc. Author's reprint from Transactions of the Ninth International Medical Congress, Vol. V.

Newport, R. I. Report of Deaths and Contagious Diseases for the Month of October, 1888.

Health in Michigan, October, 1888. Bulletin of State Board of Health, Lansing, Mich.

Twenty-first Annual Report of the Health Department of the City of Cincinnati for the year ending December 31, 1887. Byron Stanton, M. D., Health Officer.

Monthly Bulletin of the New York State Board of Health. Abstract of Reports during September, 1888.

State Board of Health Bulletin, Tennessee, for October and November, 1888. J. Berrien Lindsley, M. D., Secretary, Nashville.

Lilly's Hand-Book of Pharmacy and Therapeutics. Third edition, thoroughly revised. Eli Lilly & Co., Indianapolis, Ind.

Weekly Abstracts of Sanitary Reports, United States Marine Hospital Service. Abstracts 44, 45, 46, 47, November, 1888.

Abstract of Proceedings of the Michigan State Board of Health, October 23, 1888. Henry D. Baker, M. D., Secretary.

